# Using doxycycline for prophylaxis of bacterial sexually transmitted infections: considerations for the European Union and European Economic Area

**DOI:** 10.2807/1560-7917.ES.2023.28.46.2300621

**Published:** 2023-11-16

**Authors:** Otilia Mårdh, Diamantis Plachouras

**Affiliations:** 1 European Centre for Disease Prevention and Control (ECDC), Stockholm, Sweden

**Keywords:** doxycycline, prophylaxis, sexually transmitted infections, prevention & control, ECDC

On 28-29 June 2023, the European Network for Sexually Transmitted Infections (STI) composed of epidemiological and microbiological experts nominated by European Union/European Economic Area (EU/EEA) countries met in Stockholm, Sweden. One of the sessions in this meeting was dedicated to the use of doxycycline for the prevention of bacterial STIs and was co-chaired by a principal expert antimicrobial resistance and healthcare associated infections at ECDC and the chair of the International Union against Sexually Transmitted Infections (IUSTI) Europe. The objectives of the session were: (i) to provide an update on the evidence of efficacy of doxycycline taken before, during, or after unprotected sexual contact in reducing the incidence of chlamydia, gonorrhoea and syphilis; (ii) to present considerations on the impact of the widespread use of doxycycline on antimicrobial resistance of bacterial STIs, other pathogens and commensals, and; (iii) to discuss the potential benefits and harms of doxycycline prophylaxis at individual and public health levels, with a focus on EU/EEA context. Presentations by two invited keynote speakers were followed by an interactive discussion session among the STI Network members led by the co-chairs.

In the European Union/European Economic Area (EU/EEA), a considerable share of bacterial notifications is among gay, bisexual and other men who have sex with men (MSM). In 2021, 75% of syphilis, 62% of gonorrhoea, 19% of chlamydia and 98% of lymphogranuloma venereum (LGV) notified cases were in MSM. Trends for these STIs in MSM have been increasing in recent years, likely coinciding with the expansion of pre-exposure prophylaxis (PrEP) for HIV programmes that offer regular STI testing. There are reports that post-exposure prophylaxis (PEP) with doxycycline and other antibacterial agents, such as azithromycin and ciprofloxacin, is currently informally used by some MSM in Europe to prevent bacterial STIs [1,2].

## Doxycycline prophylaxis – evidence regarding efficacy in reducing incident STIs

In his keynote, M Unemo, University of Örebro, Sweden, provided an overview of findings from randomised clinical trials on the efficacy of doxycycline in preventing bacterial STIs when taken: (i) daily (continuously) by people engaging in sexual activity with risk of sexual transmission of bacterial STIs (pre-exposure prophylaxis) and (ii) within 24–72 hours after unprotected sexual contact (post-exposure prophylaxis).

In a randomised controlled pilot study conducted in the United States (US) in 2015, 30 MSM living with HIV infection and with high risk of STIs (defined as having had syphilis at least twice) were assigned to either take doxycycline hyclate 100 mg daily (doxy-PrEP) or receive incentive payments for remaining free of STIs. Those in the doxy-PrEP group were significantly less likely to test positive for gonorrhoea, chlamydia, and/or syphilis during the 48 weeks of follow-up (odds ratio (OR): 0.27; 95% confidence interval (CI): 0.09–0.83; p = 0.02) [3].

Further to this pilot, three open-label randomised clinical trials conducted among HIV- negative and -positive MSM and/or transgender women (TGW) with a high incidence of STIs in the last 12 months explored the efficacy of doxycycline administered as a single oral dose of 200 mg within the first 72 hours after condomless anal or oral sex (doxy-PEP).

In the French IPERGAY study in 2015, 232 HIV-negative MSM and TGW taking PrEP for HIV prevention (PrEP HIV) were assigned 1:1 to either doxy-PEP or no medication prophylaxis and followed for a median duration of 8.7 months [4]. Doxy-PEP considerably reduced the risk of incident chlamydia (hazard ratio (HR): 0.30; 95% CI: 0.13–0.70; p = 0.006) and syphilis (HR: 0.27; 95% CI: 0.07–0.98; p = 0.047). There was no significant difference between the two arms in relation to incident gonorrhoea (HR: 0·83; 95% CI: 0.47–1.47; p = 0.52). Doxycycline, 200 mg, was taken within 24 hours after condomless sexual intercourse in 83% (232/280) of events in doxy-PEP arm.

The DoxyPEP study conducted in the US, in 2022, included 501 MSM and TGW either living with HIV (PLWH cohort, n = 174) or taking PrEP to prevent HIV (PrEP cohort, n = 327) and who had at least one bacterial STI in the past year [5]. The median follow-up duration was 270 days. The study demonstrated a considerable reduction in new STI among the participants assigned to doxy-PEP (200 mg within 72 hours after condomless sex) compared with the no doxycycline arm. Moreover, doxy-PEP considerably reduced the risk of gonorrhoea, chlamydia and early syphilis in the PLWH cohort (RR: 0.43; 95% CI: 0.26–071; RR: 0.26; 95% CI: 0.12–0.57 and RR: 0.23; 95% CI: 0.04–1.29, respectively) and in the PrEP cohort (RR: 0.45; 95% CI: 0.34–0.65; RR: 0.12; 95% CI: 0.05–0.25 and RR: 0.13; 95% CI: 0.03–0.59, respectively). Of doxy-PEP users, 71% indicated never missing doxycycline within 72 hours of condomless sex.

Another trial in France (DOXYVAC), in 2022, that enrolled MSM on PrEP for HIV with a history of at least one STI in the past year indicated high efficacy of doxycycline monohydrate 200 mg taken within 24–72 hours of condomless sex vs no doxycycline over a follow-up period of 96 weeks [6]. Considerable risk reduction was seen for new episodes of chlamydia (adjusted HR (aHR): 0.11; 95% CI: 0.04–0.30) and syphilis (aHR: 0.21; 95% CI: 0.09–0.47), and a reduction by half for incident gonorrhoea (aHR: 0.49; 95% CI: 0.32–0.76). Median time (IQR) from the last sexual intercourse to doxycycline intake was 27 hours (5–33) in 80% of participants assigned to the doxy-PEP arm.

One open-label randomised trial on doxycycline 200 mg taken within 72 hours after sex compared with standard of care conducted in Kenya (2020–2022) among 449 cisgender women taking PrEP for HIV identified no reduction in bacterial STIs (any of chlamydia, gonorrhoea or syphilis) (RR: 0.88; 95% CI: 0.60–1.29), chlamydia (RR: 0.73; 95% CI: 0.47–1.13), or gonorrhoeae (RR: 1.64; 95% CI: 0.78–3.47) [7]. Doxycycline was detected in hair samples in only 44% of women in the doxycycline arm suggesting low/sub-optimal compliance.

**Key points from the presentation**

- There is evidence from three open-label randomised trials (IPERGAY, DoxyPEP and DOXYVAC) conducted among MSM and TGW with or without HIV infection and with a high incidence of bacterial STIs, that a single dose of doxycycline 200 mg taken within 24–72 hours after condomless sex can considerably (> 70%) reduce incident chlamydia and syphilis infections.
- Variable results were reported for incident gonorrhoea with a reduction of more than 50% among doxy-PEP users in two trials (DoxyPEP and DOXIVAC) but not in the IPERGAY study. Factors that could explain differences in doxy-PEP efficacy against gonorrhoea are: more doxycycline doses taken in DoxyPEP vs IPERGAY (generating a nearly steady state plasma doxycycline level due to the long doxycycline half-life), or doxycycline doses taken earlier, better adherence, and lower tetracycline resistance in the US vs France (20% vs 56%) [5].
- In contrast, one open-label trial in cisgender women with high STI incidence did not demonstrate doxy-PEP efficacy in prevention of new STIs; with low adherence, differences in anatomy and high levels of *Neisseria gonorrhoeae* resistance as potential explanatory factors.

MSM: men who have sex with men; PEP: post-exposure prophylaxis; STI: sexually transmitted infection; TGW: transgender women.

## Possible impact of widespread use of doxy-PEP on antimicrobial resistance of bacterial STIs, other pathogens and commensals

In the second keynote, C Kenyon, University of Antwerp, Belgium, indicated that while doxy-PEP use was not associated with serious adverse effects in clinical trials, there are limited data on the possible impact on antimicrobial resistance among bacteria causing STIs, commensals and human microbiota.

In the DoxyPEP trial, a higher proportion of incident gonorrhoea with tetracycline resistance was observed in the doxy-PEP group (5/13) as compared with the standard of care group (2/16), indicating a potential for selection of tetracycline-resistant *Neisseria gonorrhoeae* strains [5]. At 12 months of follow-up, *Staphylococcus aureus* colonisation in the doxycycline arm group was lower than in the standard of care group (28% vs 47%, p = 0.03), whereas an 8% absolute increase in doxycycline-resistant *S. aureus* was noted in the doxycycline arm. In in *vitro* passage experiments, doxycycline resistance emerged rapidly in *Escherichia coli* and *Klebsiella pneumoniae* [8]. A meta-analysis of results from three studies with a total of 32 cases of gonorrhoea in the tetracycline arm and 65 in the placebo arm, indicated more than double odds (OR: 2.30; 95% CI: 0.89–3.35) of minimum inhibitory concentration (MIC) ≥  2 mg/L in subjects on PEP with doxycycline or minocycline [9]. Authors of a trial of minocycline given after sexual intercourse to prevent gonorrhoea indicated that minocycline prophylaxis would probably have limited effectiveness as a public health measure because of selection of resistant gonococci [10]. Findings from a systematic review suggest that oral tetracycline use for 2–18 weeks may result in increased resistance in subgingival, gastrointestinal, and upper respiratory tract flora [11]. Another concern was the development of cross-resistance to other antimicrobial classes, through selection of genetic elements carrying resistance to multiple antimicrobial classes or through induction of efflux pumps [9]. In addition, there could be a negative impact on gastrointestinal tract microbiome diversity, potentially linked to an increased risk of inflammatory bowel disease [12].

**Key messages from the presentation**

Doxy-PEP is promising, but there is a need for additional data on potential emergence and spread of antimicrobial resistance to tetracyclines and other classes among bacteria causing STIs, but also other pathogens and commensals. More data are also needed on other potential adverse events linked to the effect on the microbiome. Such data would be useful before the use of doxy-PEP is recommended outside of study settings.

## Potential benefits and harms at individual and public health level of doxycycline prophylaxis

Points raised by meeting participants during the discussion following the keynotes included the need for clinical recommendations as some physicians are already prescribing doxy-PEP, as well as the need for guidance for community organisations on doxy-PEP messaging due to accumulating requests for advice. Furthermore, public health considerations weighing the risks and benefits of doxy-PEP on population level and as a public health intervention in the EU/EEA context would help to guide countries’ decision making on doxy-PEP implementation. Concerns were formulated regarding the impact of doxy-PEP on the detection and treatment of other STIs such as syphilis and LGV.

Modelling of the impact of doxy-PEP on long-term STI incidence and prevalence among various possible doxy-PEP user groups could help guide public health decision making. Such modelling could also take various antimicrobial resistance scenarios, at population and individual levels, into account. Furthermore, a need was expressed to map which EU countries are currently undertaking or considering doxy-PEP demonstration projects to coordinate opportunities for mutual exchange and learning. There was also a need to prepare and exchange experiences on doxy-PEP public health messaging, as well as engaging civil society in this development process.

## Conclusions

There is evidence of high efficacy of a single dose of 200mg doxycycline taken orally within 24–72 hours after condomless sex in decreasing incident chlamydia and syphilis at individual level in MSM and TGW at high risk for bacterial STIs. Doxycycline is generally safe for short-term use, however, further research is needed on the potential metabolic impact of longer-term use [13]. Results for efficacy in preventing gonorrhoea were mixed in studies conducted in Europe, possibly related to differences in resistance of *N. gonorrhoeae* between Europe and the US.

Since the meeting reported here was held, clinical guidelines were issued for consultation by the US Centers for Disease Control and Prevention advising healthcare practitioners to consider doxy-PEP in individuals at high risk for STIs (e.g. people using PrEP for HIV) as one element of a comprehensive sexual health package, accompanied by regular screening, treatment of infections, vaccinations, risk reduction counselling, awareness on benefits and known and unknown harms [14]. The European AIDS Clinical Society indicates in its 2023 guidelines update that doxy-PEP can be proposed to persons with repeated STIs living with HIV or taking PrEP for HIV on case-by-case basis [15].

Potential long-term benefits on reducing the incidence of bacterial STIs at population level and potential harms due to emergence and spread of resistance to doxycycline and other antimicrobials are still unclear. Where implemented, careful monitoring of individual and population-level resistance to doxycycline for bacterial STIs where doxycycline is first-line therapy (e.g. *Chlamydia trachomatis)* or an alternative therapy (*Mycoplasma genitalium*) will be essential, as well as monitoring of antimicrobial resistance in other pathogens.

Considering the high-quality evidence on efficacy, doxy-PEP may merit consideration as a medically supervised intervention for the prevention of bacterial STIs, especially syphilis, in some populations of MSM and TGW at high-risk for STI acquisition. If used, it should be as part of a comprehensive package of sexual health interventions, with regular screening and provision of treatment where needed and with close monitoring for individual and population-level antimicrobial resistance. Europe-wide monitoring of gonorrhea resistance trends through the Euro-GASP network, increased examination of potential doxy-PEP user needs through surveys, and exchange between countries carrying out demonstration projects to discuss practice-based evidence on effectiveness in non-trial settings will provide valuable information to countries and support monitoring and decision making on the implementation of doxy-PEP.

## *Note

This designation is without prejudice to positions on status, and is in line with United Nations Security Council Resolution 1244/99 and the International Court of Justice Opinion on the Kosovo Declaration of Independence.
